# Development of a Microneedle Swab for Acquisition of Genomic DNA From Buccal Cells

**DOI:** 10.3389/fbioe.2022.829648

**Published:** 2022-02-17

**Authors:** Yun-Seo Kim, JeongHyeon Kim, Woonsung Na, Gil-Hwan Sung, Seung-Ki Baek, Yun Kyoung Kim, Gyeong Ryeong Kim, Hae-Jin Hu, Jung-Hwan Park

**Affiliations:** ^1^ Department of Bionano Technology and Gachon BioNano Research Institute, Gachon University, Seongnam, South Korea; ^2^ Laboratory of Veterinary Virology, College of Veterinary Medicine, Chonnam National University, Gwangju, South Korea; ^3^ QuadMedicine R and D Centre, QuadMedicine Co., Ltd., Seongnam, South Korea; ^4^ Endomics, Inc., Seongnam, South Korea

**Keywords:** microneedles, swab, DNA yield, buccal mucosa, DNA purity

## Abstract

A swab is a tool for obtaining buccal DNA from buccal mucus for biological analysis. The acquisition of a sufficient amount and high quality of DNA is an important factor in determining the accuracy of a diagnosis. A microneedle swab (MN swab) was developed to obtain more oral mucosal tissues non-invasively. Eight types of MN swabs were prepared with varying combinations of patterns (zigzag or straight), number of MNs, intervals of MNs, and sharpness of tips. When MN swab was applied up to 10 times, the tissue amount and DNA yield increased compared to commercial swabs. A zigzag pattern of microneedles was found to be more efficient than a straight pattern and increasing the number of microneedles in an array increased the DNA yield. The MN swab collected about twice the DNA compared to the commercial swab. In an *in vivo* test using mini pigs, the lower cycle threshold values of mucosal samples collected with MN swabs compared to samples collected with commercial swabs indicated that a greater amount of DNA was collected for SNP genotyping. A polymer MN swab is easy to manufacture by a single molding process, and it has a greater sampling capacity than existing commercial swabs.

## Introduction

For testing various diseases such as diabetes, hyperlipidemia, and infectious diseases, a blood collection method has been widely used because it provides high quality and large amounts of DNA (Grady et al., 2014; De Sousa et al., 2017; Blauwkamp et al., 2019). However, this method has several drawbacks, including pain caused by the syringe, need for medical expertise, high cost, and use of biohazardous material. Thus, a buccal swab has been suggested to solve these limitations of blood collection (García-Closas et al., 2001; Saftlas et al., 2004; Frantz Burger et al., 2005; Herráez and Stoneking, 2008; Van Wieren-De Wijer et al., 2009).

The oral cavity is an ideal place to obtain biological samples such as microorganisms, viruses, protein, and DNA material (Frantz Burger et al., 2005; Schulz et al., 2013; Yu et al., 2017; Kam et al., 2020). The role of oral microbiota in various chronic diseases is reported by collecting microorganisms present in the mucous membrane of the oral cavity. Recently, it was announced that the SARS-CoV-2 virus is particularly distributed in the oral mucosa, so the oral mucosa is a suitable source for detecting this virus (Kam et al., 2020).

By monitoring biomarkers indicating human genetic damage from buccal cells, cancer risk and degenerative diseases can be predicted (Oßwald et al., 2003; Proia et al., 2006; Holland et al., 2008). Interest in a direct-to-consumer (DTC) test of a person’s genome is increasing (Su, 2013). Methods of obtaining samples from the oral cavity have been used in various fields, including genomics, proteomics, metabolomics, microbiomes, and epigenomics (Theda et al., 2018). There are several methods for collecting buccal cells, such as a cytobrush and mouthwash (García-Closas et al., 2001; Le Marchand et al., 2001), but the most commonly used method is a buccal swab because it is non-invasive, time-saving, and cost-effective (Saftlas et al., 2004; Frantz Burger et al., 2005; Van Wieren-De Wijer et al., 2009).

Commercial swabs are made of a variety of materials, including cotton, polyester, rayon, and nylon. The method of discharging samples from the swab depends on the structure of the swab material (Dube et al., 2013; Bruijns et al., 2018; Zasada et al., 2020). With a cotton or rayon swab, for example, the fiber is wrapped around the shaft of the swab. Although this kind of swab is the most widely and routinely used, it may negatively affect DNA analysis using polymerase chain reaction (PCR) because they can leave the cotton fibers or other impurities in the reaction solution. Commercial swabs require more amount of sample to be obtained because of contaminants such as bacteria or food present on the mucosal surface (Brownlow et al., 2012; Bruijns et al., 2018). The recovery rate from using this kind of swab is slow because of the wrapped structure of the fiber (Adamowicz et al., 2014). For flocked swabs made of nylon, on the other hand, short fibers are attached to the shaft and they are not wrapped-around. This structure is known to be more advantageous in sampling efficiency or recovery than conventional cotton swabs, but it can leave swab material on the rough surface (Brownlow et al., 2012).

Microneedles (MNs) have been used in cosmetics and medicine as one of the transdermal delivery systems because they are less invasive and cause less pain. The microneedle structure penetrates the stratum corneum of the skin barrier to deliver active ingredients into the skin (Kim et al., 2012). As reported in a previous study, when a MN array 280 μm in length was applied to the forearm, the value of a visual analogue scale (VAS) was measured at an average of 0.2 cm (out of 10 cm), and there was no pain (Jeong et al., 2017). Buccal mucosa is composed of about 40–50 cell layers (Harris and Robinson, 1992), and the average thickness of the epithelial layer of human buccal mucosa is 600 µm (Di Stasio et al., 2019). Blood vessels in buccal mucosa are usually located deeper than 600 μm (Oh et al., 2021). Therefore, short MN arrays (less than 300 μm) typically do not reach blood vessels. Clinical studies have reported that MN arrays 200 μm in length cause no discomfort (Xie et al., 2005).

**FIGURE 1:**
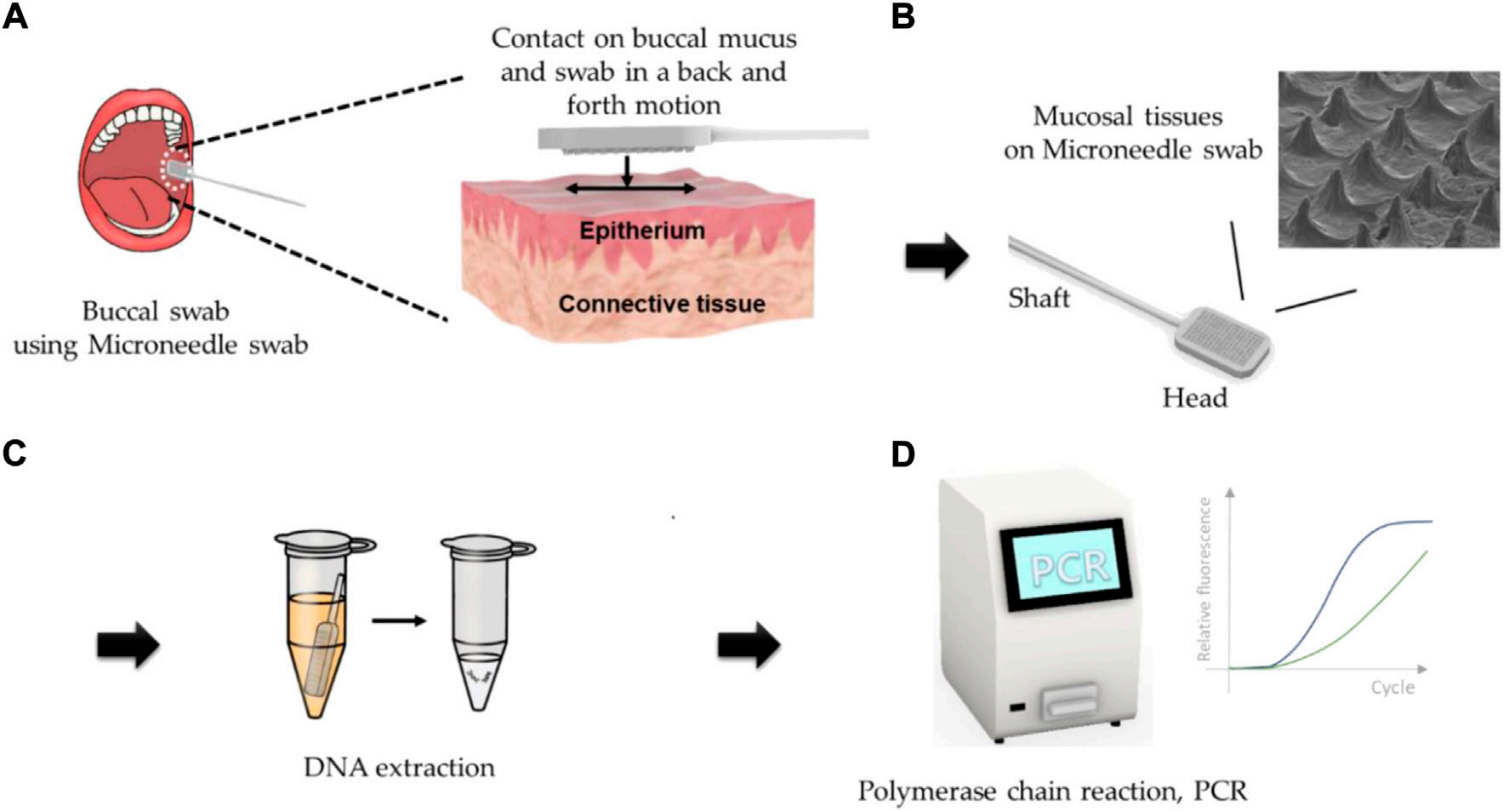
Graphical descriptions of operation of microneedle swab **(A)** The upper layer of buccal mucosal tissue was obtained by swabbing the surface of buccal mucosa using the microneedle swab. **(B)** Obtained sample is located at the surface of the microneedle. **(C)** Sample is released to lysis buffer quickly and DNA is extracted by swab lysis protocol. **(D)** Desired genetic information can be obtained through PCR process.

In this study, MNs were applied for the purpose of obtaining tissue samples more effectively from buccal mucosa. The microneedle swap system consists of the followings as shown in Figure 1, **I)** The upper layer of buccal mucosal tissue was obtained by swabbing the surface of buccal mucosa using microneedle swab. The thickness of the epithelium of human buccal is about 600 μm, thus microneedles of less than 250 μm in length is used for less pain and safety, Figure 1A, **II)** Obtained sample is located at the surface of the microneedle swab, Figure 1B. **III)** and Sample is released to lysis buffer quickly and DNA is extracted by swab lysis protocol, Figure 1C. **IV)** Desired genetic information can be obtained through PCR process, Figure 1D. MN swabs with various geometries were fabricated from biodegradable or non-degradable medical polymers, and the MN swabs were optimized to achieve best performance. The MN swabs were manufactured using a polymeric molding process for easy fabrication and cost-effectiveness. In the present study, the efficacy of the MN swab was verified by comparing it with two commercial swabs in *ex vivo* and *in vivo* experiments.

## Materials and Method

### Preparation of Microneedle Swab

A MN swab consists of a head containing the microneedles and a handle. The head is a rectangle with rounded corners and measures 9 mm wide by 15 mm long by 2 mm thick. Pyramid-shaped microneedles (250 μm high with a base length of 150 μm or 200 μm) are placed on the head. In addition, a hole is made at the end of the head to connect the handle. Eight types of heads with different patterns, numbers of microneedles, spacing, and tip sharpness were prepared (see summary in Table 1).

**TABLE 1:** Information on eight variations of microneedles attached to a swab head.

Model No.^a^ (pattern-number- interval- sharpness)	Pattern	Number of MNs [ea]	Interval between MNs [µm]	Sharpness of MN [µm]
S-N4-I2-S20	Straight	496	200	20
S-N2-I2-S20	Straight	248	200	20
S-N2-I4-S20	Straight	248	400	20
S-N3-I3-S7	Straight	338	280	7
Z-N4-I2-S20	Zigzag	496	200	20
Z-N2-I2-S20	Zigzag	248	200	20
Z-N2-I4-S20	Zigzag	248	400	20
F	Flat	—	—	—

a S, straight; Z, zigzag; N, number of microneedles; I, interval; S, sharpness from model number.

**FIGURE 2:**
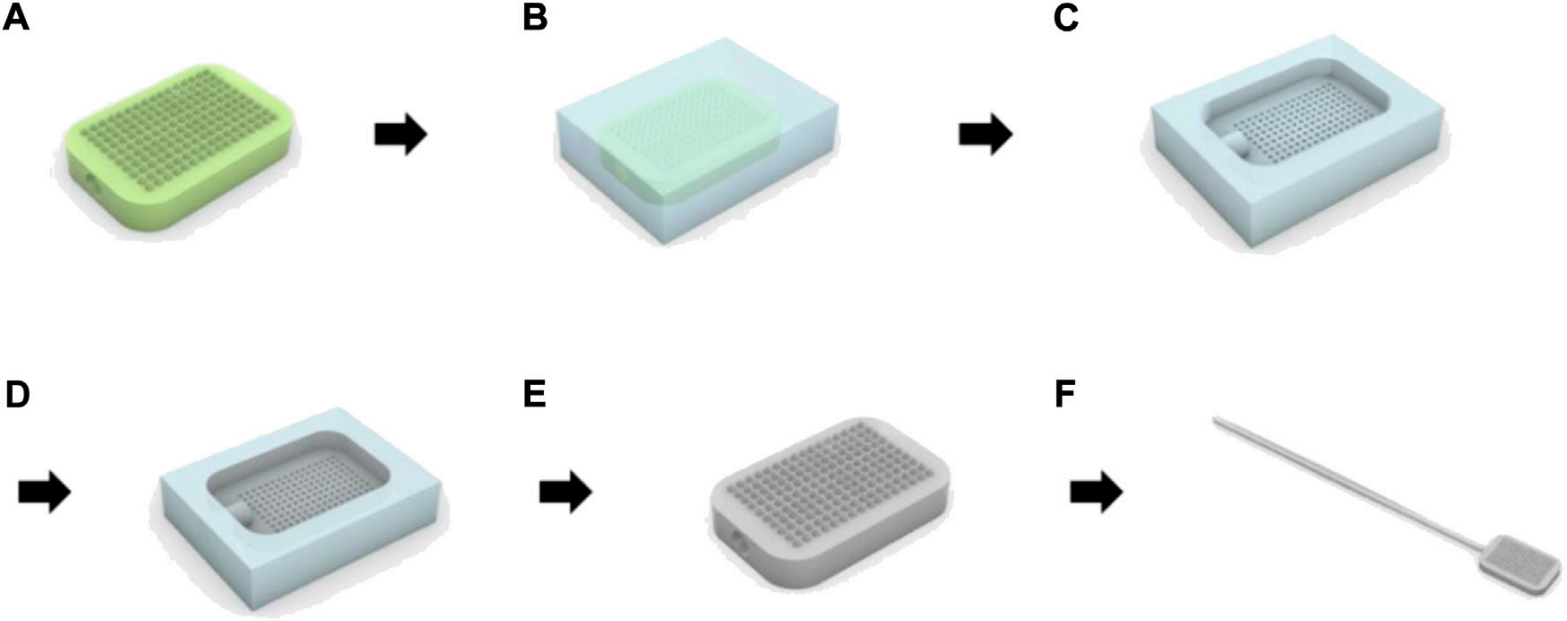
Manufacturing process of microneedle swab using polymer melt micromolding. **(A)** Production of the master structure of the head with the microneedles by micro-milling or 3D printer. **(B)** Fabrication of PDMS (polydimethylsiloxane) mold. **(C)** Removal of the cured PDMS mold from the master structure. **(D)** Preparation of replicate using polymer micromolding process. **(E)** Removal of polymer replicates from PDMS mold. **(F)** Integration of the head with handle.

As shown in Figure 2A, a micro-milling process and three-dimensional (3D) printing were used to fabricate the master structures of the swab head. To obtain a polydimethylsiloxane (PDMS, Sylgard 184, Dow Corning, MI, United States) mold from the master structure (Figure 2B), an uncured PDMS mixture was poured on the master structure and cured at 70°C for 1 h (Figure 2C). Pellets of polylactic acid (PLA), cyclic olefin copolymer (COC), or polycaprolactone (PCL) were placed on the PDMS mold. The mold was then melted at −90 kPa vacuum pressure and 195°C in a vacuum oven (VOS-301, EYELA, Tokyo) (Figure 2D). Replicates were released from the mold after cooling (Figure 2E). The head of the MN swab was connected to a 12-cm-long polypropylene (PP) stick. (Figure 2F).

### Mechanical Properties of Microneedle Swab

Microneedle swabs were manufactured out of three polymers (polylactic acid (PLA), Cyclic olefin copolymer (COC), Polycaprolactone (PCL)), and the mechanical strength for successful swabbing with each polymer was investigated. Porcine oral mucosa with 400 μm thick epithelium of porcine buccal tissue of thickness 5 mm was fixed on a 1-cm-thick wood plate using a pin, and the plate was placed on a scale. After the microneedle head came into contact with the porcine oral mucosa, a microneedle swab was applied 10 times [5 repetitions (back and forth)] with a force of 200–300 g. Thereafter, the deformation of the microneedle tips of the swab was observed using scanning electron microscopy (SEM, JSM-7001F, JEOL Ltd., Tokyo, Japan).

### *Ex Vivo* Efficacy of Microneedle Swab

The amount of collected tissue, DNA yield, and DNA purity achieved as a result of applying the microneedle swab were measured using the porcine oral mucosa. These results were compared with the results obtained by using the commercial swabs.

#### Observation of the Mucosal Tissue Collected on the Swab Surface Using SEM and EDS (Energy Dispersive X-Ray Spectrometer)

To observe the mucosal tissue collected on the surface of the MN swab, the *ex vivo* porcine oral mucosa was swabbed with a S-N3-I3-S7 swab. After swabbing, the samples were dried in a desiccator at room temperature for 10 h. Samples before and after swabbing were coated with platinum for 120 s using a sputter coater. The surface of the samples was observed for morphology using SEM. Chemical analysis of the sample surface was performed using the Energy-Dispersive Spectroscopy (EDS, JEOL Ltd.) mounted on the SEM, which detected nitrogen and thus confirmed the presences of mucous tissue.

#### DNA Extraction Protocol for Microneedle Swab

Porcine mucosa was purchased from CRONEX (Seoul, South Korea). The mucosal surface was cleaned by washing the surface with phosphate buffered saline (PBS). After fixing the oral mucosa on the plate, the microneedle head was brought into contact with the oral mucosa and swabbed 10 times. An intact MN swab not used for swabbing was set as a negative control.

DNA from the sample was extracted using a QIAamp DNA mini kit (Qiagen, Hilden, Germany). Two extraction protocols were compared: 1) DNA extraction from buccal swabs (swab Protocol), and 2) DNA extraction from tissue (tissue Protocol).

**FIGURE 3:**
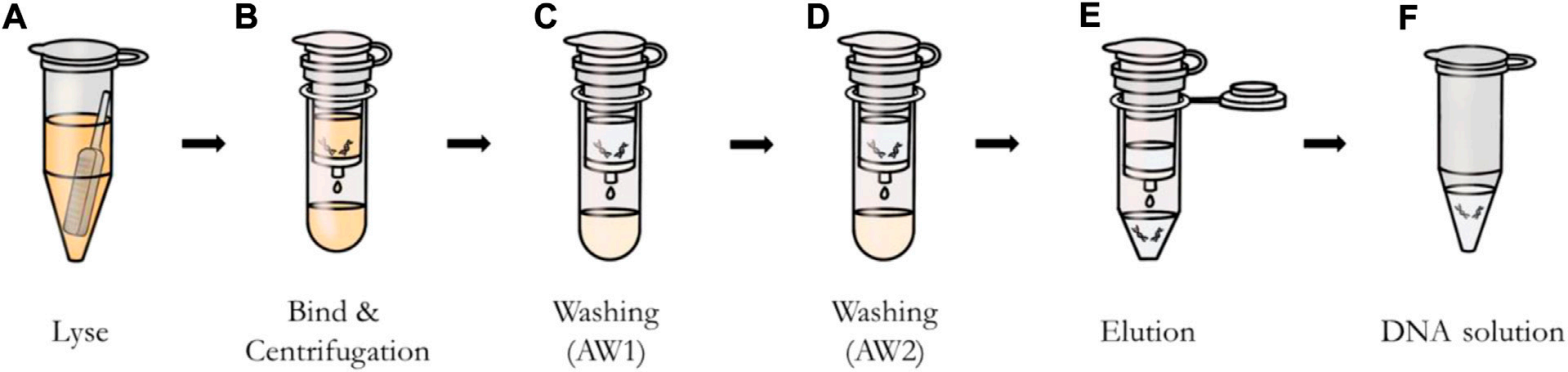
DNA extraction process using QIAamp DNA mini kit. **(A)** Lysis of sample from swab. **(B)** Bond of DNA to the column by applying and centrifuging the solution through the column. **(C)** and **(D)** Removal of impurities except DNA with buffers AW1 and AW2. **(E)** Elution of DNA bound to the column using buffer AE. **(F)** Extracted DNA solution.

The buccal swab protocol is as follows. The swab was put in the tube and 600 μl of PBS was added (Figure 3A). Then 20 μl of proteinase K and 600 μl of buffer AL were added to sample solution and mixed by vortexing. After incubation at 56°C, 600 μl of ethanol was added. The entire solution was applied to the spin column and centrifuged at 8,000 rpm for 1 min (Figure 3B). Additional centrifugation was then performed at 8,000 rpm after adding 500 μl of buffer AW1 (wash) to the sample solution (Figure 3C), and centrifugation at 14,000 rpm was applied after adding buffer AW2 to the sample solution (Figure 3D). Finally, as shown in Figure 3E, 50 μl of elution buffer was applied, incubated for 1 min, and centrifuged at 8,000 rpm to obtain DNA (Figure 3F).

For the tissue protocol, the swab was placed in a solution of 420 μl of PBS, 180 μl of buffer ATL (tissue lysis), and 20 μl of proteinase K. After incubation at 56°C, 600 μl of buffer ATL was added. After an additional incubation at 70°C for 10 min, 600 μl of ethanol was added, and the entire solution was applied to the column as a swab protocol. After that, the washing and elution procedure was the same as in the swab protocol.

The DNA concentration was measured at a wavelength of 260 nm using a NanoDrop^®^ ND-1000 spectrophotometer (Nanodrop Technologies Inc., NC, United States). The concentration was measured at various incubation times of 10 min, 30 min, 1, 3, and 6 h, and the DNA yield at 6 h was set to the maximum value. The DNA amount was observed over time and measured for the same mucosa.

#### Comparison of Tissue Amount According to Number of Swabbings and Head Geometry

The amount of tissue collected after various swabbings (i.e., swabbing 2, 5, 10, 15, and 20) was measured using the microneedle swab (Z-N4-I2-S20) and the commercial Isohelix^®^ swab. In addition, for comparison according to the type of head, the DNA yield from a total of eight types of MN swabs, including the flat head swab, was compared at the fixed swabbing number of 10 times.

The amount of tissue collected was calculated by measuring weight change. Namely, the weight of the swab before swabbing was measured using a micro-scale, and after swabbing, the swab was dried in a desiccator for 10 h. Then the weight of the dried sample was measured. All experiments were repeated three times.

In order to observe the difference in number of columns by swabbing between the zigzag pattern and straight pattern of microneedles, microneedle swabs were swabbing porcine skin with 200–300 g of force once. Then trypan blue soluion (0.5% (w/w)) was dropped on the swabbed surface for 30 s and removed. The number of stained lines on the porcine skin was observed by using optical microscope (Leica M125, Wetzlar, Genmany).

Porcine mucosa was swabbed 10 times with microneedle swab *ex vivo*. The porcine mucosal tissue was freeze-dried using a freeze dryer (LP03, IlShin BioBase, Korea) and the morphology of surface was observed at ×100 and × 500 by SEM (30 tilt angle). The depth of valley of intact buccal mucosa surface and swabbed surface was measured from images, and these values were compared.

### Comparison of Microneedle Swab With Commercial Swab

#### Comparison for Release Efficiency

The sample release efficiency of the microneedle swab was compared with that of the commercial Isohelix^®^ and Copan eSwab^®^ swabs. To prepare the DNA solution to be applied to the swab head, the oral mucosal tissue of the pig was extracted using the “DNA Purification from Tissues” protocol of the QIAamp DNA mini kit. Then, 40 μl of DNA solution was dropped on each swab head [MN swab, rayon swab (Isohelix^®^), and nylon flocked swab (Copan eSwab^®^)] with a pipette. Each swab head was cut off and placed in a 5 ml tube filled with 500 μl of buffer and vortexed for 1 min. Release efficiency (RE) was calculated as follows.

$$RE=\;\frac{V_{\mathrm{buffer}}\left[sample\right]}{V_{\mathrm{applied}}\left[origin\right]}100\;\%$$

The ratio of the amount of DNA released into the buffer compared to the amount of DNA applied to the swab head was expressed as a percentage. *Vapplied* in the denominator is the volume (40 μl) of the DNA solution applied to the swab head, and [origin] is the concentration of the DNA solution in the application solution. *Vbuffer* is the lysis buffer volume, 500 μl, and [sample] is the DNA concentration released into the buffer (Bruijns et al., 2018).

#### Comparison for DNA Yield and Purity

The swabbing method is the same as the *ex vivo* test described above. S-N3-I3-S7 microneedle swabs and commercial swabs (Isohelix^®^ swab made of rayon and Copan eSwab^®^ made of flocked nylon) were compared for DNA yield and purity with a NanoDrop. The value of absorbance at 260 nm and the ratio of absorbance 260/280 were measured. All experiments were repeated three times.

### *In vivo* Efficacy Study of Microneedle Swab

#### Sample Collection

All studies were approved by Institutional Animal Care and Use Committees (IACUC) at the Chonnam National University (CNU IACUC-YB-2021-95). Ten mini-pigs (18–20 kg, ages: 6–7 months, XP Bio, Korea) were divided into two groups of five each, and oral samples were collected from the mucosa of the pigs using a microneedle swab (Z-N4-I2-S20) (Group A) and a nylon flocked swab (Copan eSwab^®^) (Group B). The number of swabbings was 10 times for both groups. The swabs with collected samples were placed in 5 ml tubes (Eppendorf) and stored at 4°C until DNA extraction. For comparison with a blood sample as a standard, blood was also collected from each mini-pig, placed in an tube, and stored at 4°C.

#### DNA Analysis

The DNA of each mini-pig was extracted using the QIAamp DNA mini kit. With the blood sample, we followed the “DNA purification from blood or body fluids” protocol, and with the swab we followed the “DNA purification from buccal swabs” protocol as before. Briefly, to extract DNA from blood, 200 μl of blood was mixed with 20 μl of proteinase K and 200 μl of buffer AL. Then, the protocol was carried out in the same way as the swab protocol. The concentration of DNA solution extracted from blood and swab was measured with a NanoDrop. SNP genotyping was performed to confirm that the amount of DNA collected from the MN swab was sufficient compared to blood using the QuantStudio 3 real-time PCR device (Life Technologies, CA, United States). For the SNP genotyping test, Master Mix, a premixed solution containing necessary ingredients, was used (SFC SNP Genotyping Master Mix). The volume of DNA solution was 5 μl. For the SNP genotyping test, two probes are provided; one probe is labeled with VIC dye and the other with FAM dye. Alleles can be distinguished by competitively binding the dyes attached to the two allele-specific primers, and each wavelength of the different dyes was independently detected by a real-time PCR device. The reaction mixture in the SNP genotyping test is shown in Table. 2.

**TABLE 2:** Assay setup for the SNP genotyping test.

Component	Volume (μl)	Final concentration
SFC SNP Genotyping Master Mix (2X)	12.5	1X
SFC SNP Assay Mix (40X)	0.625	1X
DNA Template	5	10 ng∼/rnx
Distilled water	6.875	
Total volume	25	

The thermal cycle conditions comprised a pre-denaturation step for 3 min at 95°C followed by 50 cycles: 95°C for 10 s, 60°C for 30 s. Allelic discrimination plots for genotyping were generated using QuntiStudio3 software.

### Statistics

A two-tailed Student’s t test (*α* = 0.05) was used to compare two groups, and ANOVA was used to compare multiple groups. A *p*-value less than 0.05 (typically ≤0.05) is considered statistically significant.

## Results and Discussion

### Geometries of Microneedle Swab

**FIGURE 4:**
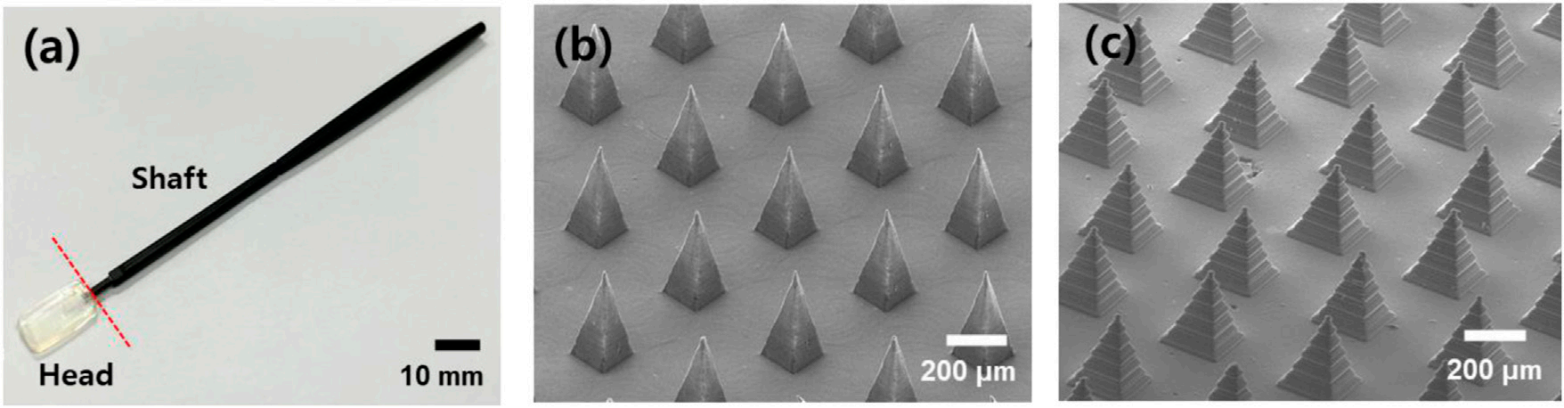
**(A)** Optical image of the microneedle swab (scale bar, 10 mm). The swab consists of a head and a handle. **(B)** SEM image of MN from micro-milling (S-N3-I3-S7). **(C)** MN prepared from 3D printed master structure (Z-N4-I2-S20) (scale bars, 200 μm).

The handling stick was inserted into a hole in the swab head and a microneedle swab was prepared as shown in Figure 4A. The solid microneedles were placed on the head in an area 15 × 9 × 2 mm. Figures 4B–C are SEM images of the PLA microneedle array from the master structure, fabricated by micro-milling and 3D printing, respectively. As shown in Figures 4B–C, the two kinds of microneedles used in this study were of different sharpness (micro-milling, 7 μm; 3D printer, 20 μm). However, since various types of heads could be easily prepared using a 3D printer, the head master structures (except S-N3-I3-S7) were manufactured with a 3D printer to compare the efficacy by other factors except sharpness.

**FIGURE 5:**
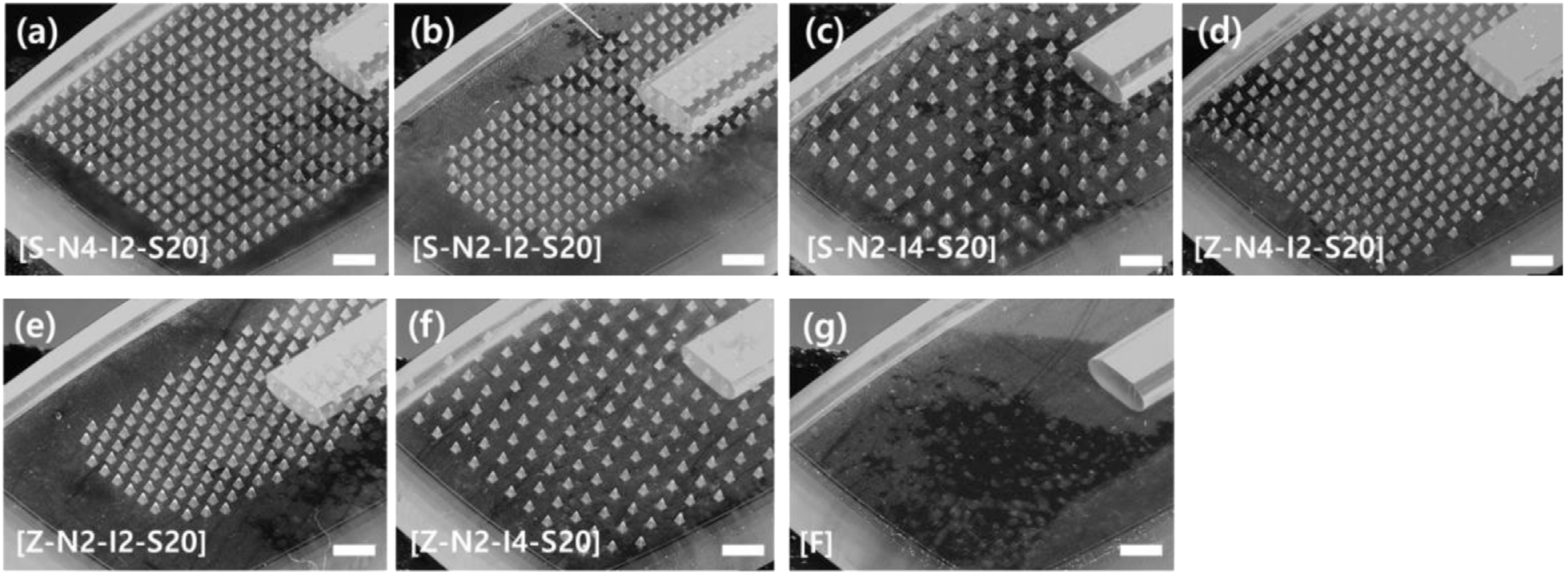
Optical microscope image of seven types of microneedle swab heads according to the number, spacing, and pattern of microneedles (scale bars, 1 mm). **(A)** S-N4-I2-S20: Straight-No. 496 ea-Interval of 200 μm-sharpness of 20 μm. **(B)** S-N2-I2-S20: Straight-No. 248 ea-Interval of 200 μm-sharpness of 20 μm. **(C)** S-N2-I4-S20: Straight-No. 248 ea-Interval of 400 μm-sharpness of 20 μm. **(D)** Z-N4-I2-S20: Zigzag-No. 496 ea-Interval of 200 μm-sharpness of 20 μm. **(E)** Z-N2-I2-S20: Zigzag-No. 248 ea-Interval of 200 μm-sharpness of 20 μm. **(F)** Z-N2-I4-S20: Zigzag-No. 248 ea-Interval of 400 μm-sharpness of 20 μm. **(G)** Flat head without microneedles.

Seven types of MN head structures were prepared according to the pattern, number, and spacing between MNs. Seven types of head structures were prepared for MNs with a sharpness of 20 μm (Figure 5). When a head with MN sharpness of 7 μm was included, eight types of heads were prepared. Two array patterns were used: straight arrays and zigzag arrays. Heads with three different numbers of MNs were prepared: 248, 338, and 496. The three different spacings were 200, 280, and 400 μm. The serial number of each sample was expressed as pattern-number-spacing-sharpness (Figures 5A–F). For example, S-N4-I2-S20 represents a head with a straight array, 496 MNs, an interval of 200 μm, and a tip sharpness of 20 μm. As a negative control, a head with a flat surface without a microneedle was used (Figure 5G). In all cases, the height of the microneedles was 250 μm.

### Mechanical Property of Microneedle Swab

Microneedle swabs were fabricated from three kinds of polymers with different mechanical strength, and the degree of deformation of the microneedles was compared after swabbing on the mucous membrane. All three polymers are medical grade polymers. COC is a non-degradable medical polymer, and PCL and PLA are biodegradable medical polymers. Medical polymers were selected as microneedle swab material because clinical research and medical device licensing aspects of microneedle swabs were taken into consideration. Young’s modulus and yield strength of PLA are, respectively, 3.9 GPa and 85 Mpa (Orue et al., 2016; Jeong et al., 2020), of COC are 3.2 GPa and 89.3 Mpa (O’neil et al., 2016; Gopanna et al., 2018), and of PCL are 0.28 GPa and 33.0 Mpa (Avella et al., 2000). The mechanical strength of PLA and COC is similar, whereas the mechanical strength of PCL is relatively lower than that of the other two polymers. In the deformation comparison study, S-N3-I3-S7 with a sharpness of 7 µm instead of 20 µm was used in order to closely observe the mechanical deformation according to the type of polymer.

**FIGURE 6:**
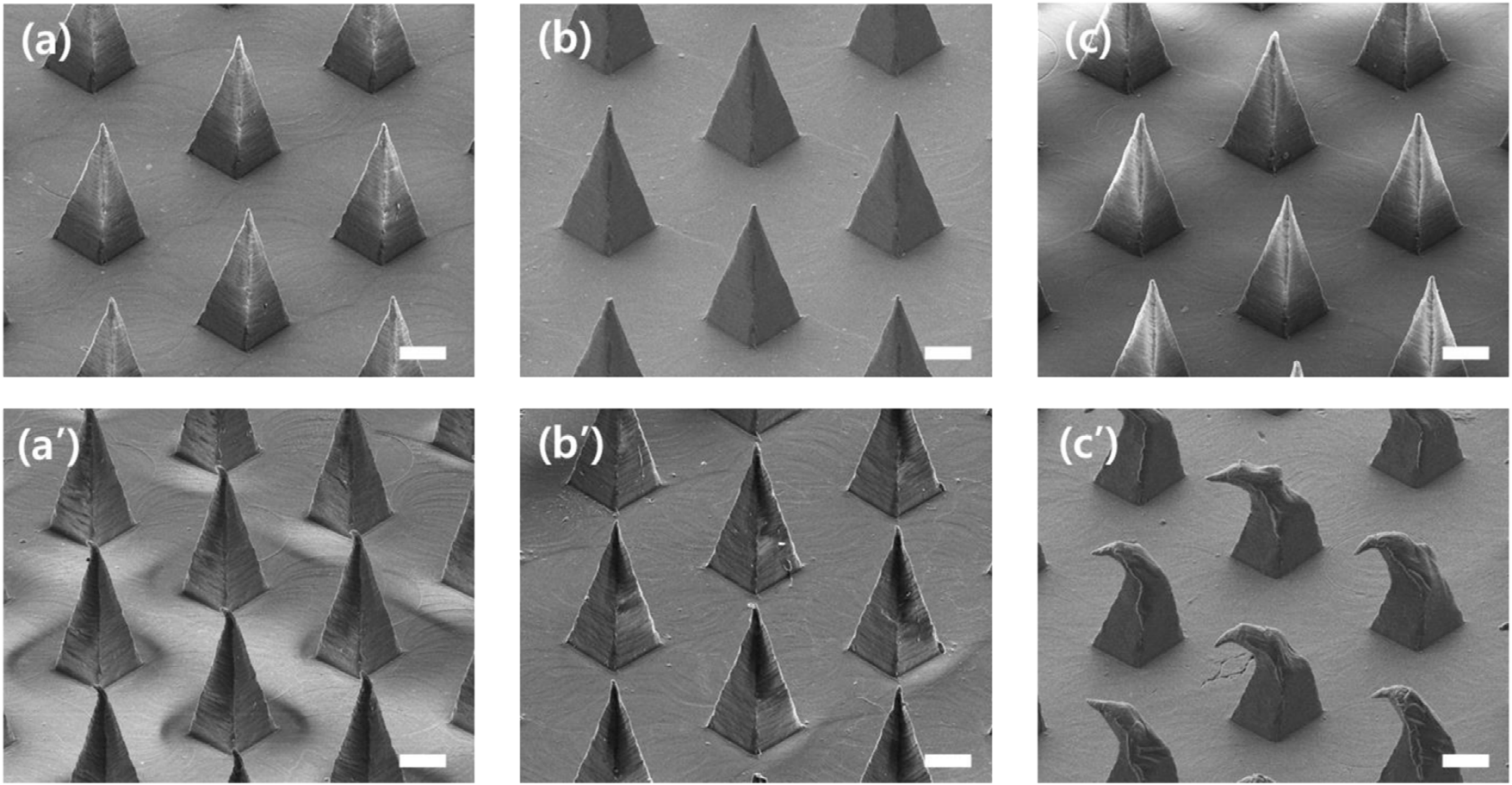
SEM image of microneedles before and after swabbing with swab S-N3-I3-S7 **(A,B)** Before and after swabbing with polylactic acid (PLA) MN **(C,D)** Before and after swabbing with cyclic olefin copolymer (COC) MN. **(E,F)** Before and after swabbing with polycaprolactone (PCL) MN (scale bars, 100 μm).

As shown in Figure 6, the tips of PLA and COC MNs had almost no mechanical deformation after swabbing. However, because of the low mechanical strength of PCL, the tip end was bent after swabbing. Therefore, PLA and COC have sufficient mechanical strength to prevent deformation from occurring during the sampling process.

### Efficacy of Microneedle Swab in *ex vivo* Test

#### Surface Images of Collected Samples on Swabs

**FIGURE 7:**
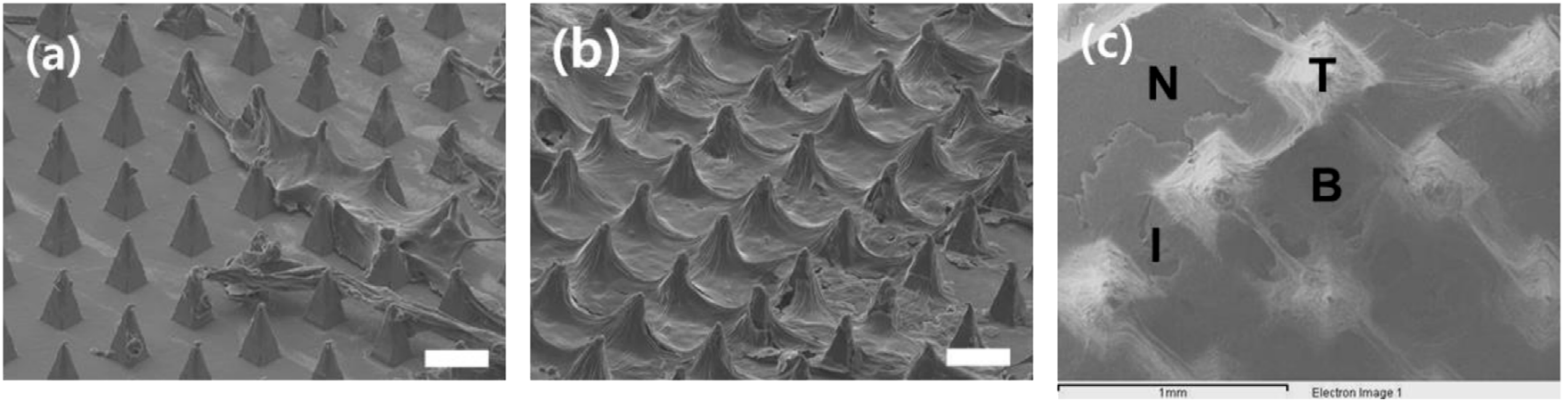
SEM image of the surface of S-N3-I3-S7 microneedle swabs on the *ex vivo* pig oral mucosa after swabbing **(A)** 5 times and **(B)** 15 times (scale bars, 300 μm). **(C)** Detection position by EDS. T is the tip part of the needle, I is the interval part between needles, B is the base part of the array with the sample, and N is the part without the sample.

Surface images of the S-N3-I3-S7 microneedle swab were obtained by SEM after 5 and 15 times of swabbing, respectively. As shown in Figure 7A and Figure 7B, the dried sample was distributed on the swab. As the number of swabbing increased, more material was collected around the microneedle tips.

An EDS was used to check whether nitrogen was present in the sample, which would confirm the presence of mucous tissue. Figure 7C is an image of the sample on the MN swab, and it shows the position detected by the EDS. Our results indicate that the atomic percentages of nitrogen on the tip(T), in the interval(I), and at the base(B) were 21.80, 24.29, and 24.17%, respectively. On the other hand, the percentage of nitrogen in the negative control group(N) was 0%. The dried sample on the MN swab contained tissue that included nitrogen.

The depth of the valley was about 10 μm for intact buccal mucosa and 20 μm after swabbing 10 times with microneedle swab as shown in Supplementary Figure S3. The microneedle swab formed low-depth valleys on the mucosal surface compared to 250 μm length of microneedles. In clinical studies with wound in the oral mucosa, mucous membranes recovered faster than skin, induced minor scars, and reported rapid and transient inflammation. The reason of rapid recovery is suggested to be due to a distinct fibroblast phenotype, the presence of bacteria that stimulate wound healing and the moist environment and growth factors present in saliva (Larjava et al., 2011), (Desjardins-Park et al., 2019). Therefore, the oral tissue provides a favorable recovery environment compared to the skin. However, these safety points including infection will be studied through clinical studies.

#### DNA Extraction From Microneedle Swab

**FIGURE 8:**
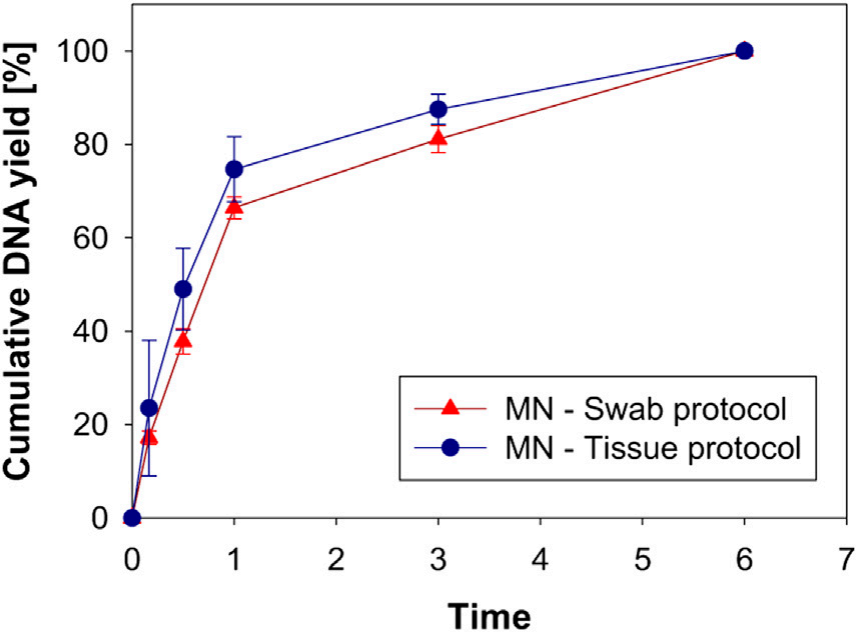
DNA yield according to lysis time and extraction protocol.

DNA yield was measured according to the incubation time after the swab protocol or the tissue protocol was applied. DNA yield at an incubation time of 6 h was set to 100%, and DNA yield at each time point was expressed as a percentage. Figure 8 shows the cumulative DNA yield according to the extraction protocol and incubation time. The cumulative DNA yield from to the two protocols was not significantly different, although the average DNA yield of the tissue protocol was slightly higher. Also, there was no difference in lysis time for either the swab protocol or the tissue protocol. Considering that the tissue protocol requires a second incubation procedure, the simpler swab protocol seems more suitable for MN swabbing. To extract DNA, collected samples should be released from the swab, and the cell membrane and the nuclear membrane of the sample are broken through the lysis process at 56°C (Shehadul Islam et al., 2017). When the DNA yield at 6 h was 100% using the swab protocol, 37.8 ± 4.7% of DNA was extracted after 30 min of lysis, and 66.4 ± 4.1% was extracted after 1 h of lysis. In the ongoing experiment, the incubation time was set to 1 h, considering DNA yield and short analysis time.

#### Effect of Tissue Amount According to Number of Swabbings and Geometry of Microneedle Swab Head on DNA Yield

**FIGURE 9:**
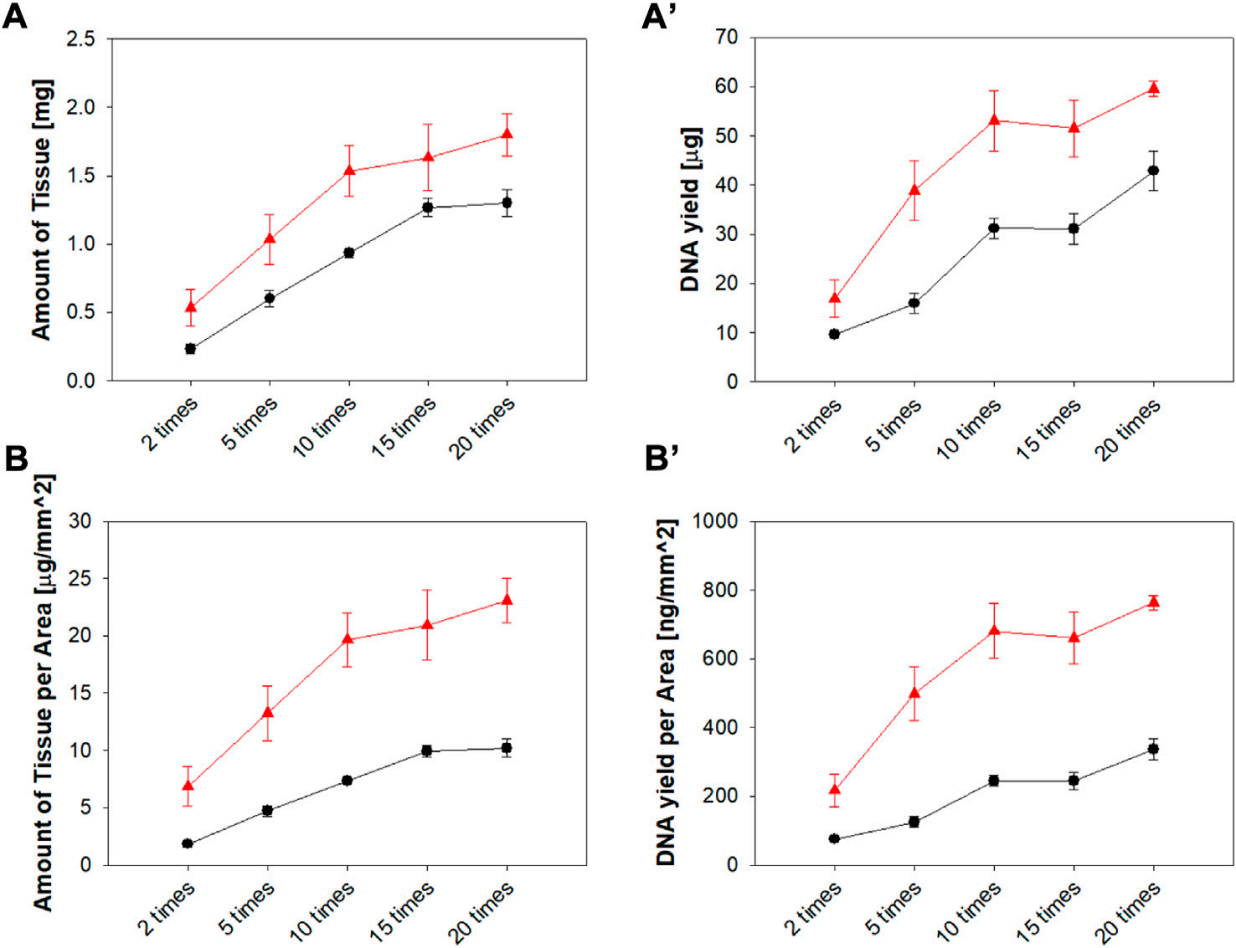
**(A)** Tissue amount and **(A')** DNA yield according to the number of swabbings (*ex vivo*) of the pig mucosa with Z-N4-I2-S20 microneedle swab and rayon swab (Isohelix^®^). **(B)** Tissue amount and **(B’)** DNA yield per unit area (▲: MN swab, ●: rayon swab) (n = 3).

Commercial swabs require vigorous swabbing to obtain sufficient tissue and DNA samples.(Walker et al., 1999; Mcmichael et al., 2009). The amount of tissue and DNA obtained with a MN swab (Z-N4-I2-S20) was observed according to the number of swabbings and was compared with the amount of tissue, DNA, and number of swabbings required by the commercial swab (Isohelix^®^). Figure 9 shows the greater amount of tissue and DNA obtained per unit area using the microneedle swab compared with the amount obtained using the commercial swab.

As the number of swabbings increased, the amount of tissue and DNA obtained increased. The increase in tissue mass and DNA yield become gradual after 10 swabbings. With the MN swab, 10 swabbings is the optimal number when both user convenience and efficacy are considered together. Based on this result, the number of swabbings was set to 10 times for the *in vivo* experiment.

The MN swab collected more tissue and DNA than the rayon swab (Isohelix^®^) for all times of swabbing. In particular, the superiority of the MN swab was more evident when the unit area of the head was considered: the head area of the MN swab was 78 mm^2^ and the area of the rayon swab of 127 mm^2^. In addition, the same amount of tissue was obtained with fewer swabbings with the MN swab compared to the Isohelix^®^ swab. This resulted in improved convenience and fewer test errors caused by insufficient collection of DNA.

**FIGURE 10:**
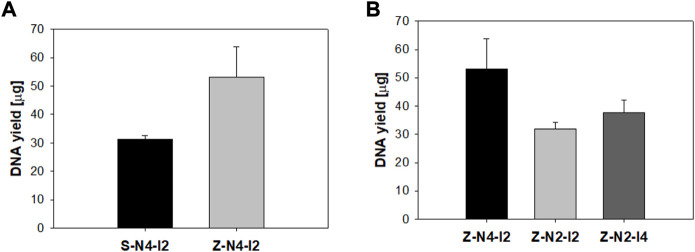
**(A)** Comparison of DNA yield according to microneedle pattern (S: straight, Z: zigzag). The sharpness of both models equals 20 μm (S-N4-I2-S20, Z-N4-I2-S20). **(B)** Comparison of DNA yield according to the number of microneedles (Z-N4-I2, Z-N2-I2) and the interval between microneedles (Z-N2-I2, Z-N2-I4) (n = 3).

To optimize the design of the microneedle head, the DNA yield was compared according to the geometry of the head. First, the DNA yield was observed according to the pattern of the microneedles by using 2 MN swabs of S-N4-I2-S20 and Z-N4-I2-S20 having the same number and spacing of microneedles. As shown in Figure 10A, the average values of DNA yield obtained from the zigzag array were higher than those from the straight array (*p* < 0.05). The DNA yield using the zigzag pattern increased because the columns formed by the first row and those formed by the second row are different. The number of columns formed by microneedle swab with zigzag pattern was twice that with microneedle swab with straight pattern after swabbing the porcine skin once as shown in Supplementary Figure S2 in supplement. S-N4-I2-S20 formed 16 columns on the mucus by single swabbing, whereas Z-N4-I2-S20 formed 32 columns by single swabbing.

Next, efficacy was compared according to the spacing and number of microneedles of the MN swab with the same pattern of zigzag (Figure 10B). The DNA yield obtained from Z-N4-I2 (496, 200 μm) was highest (2-way ANOVA, *p* = 0.045). The number of microneedles was an important factor in determining efficacy, and the microneedles played an important role in obtaining samples from the mucous surface. Therefore, Z-N4-I2-S20 was recommended for geometries of the MN swab because it had the largest number of microneedles and a zigzag pattern.

#### Comparisons of Morphology Between Commercial Swabs and Microneedle Swab

**FIGURE 11:**
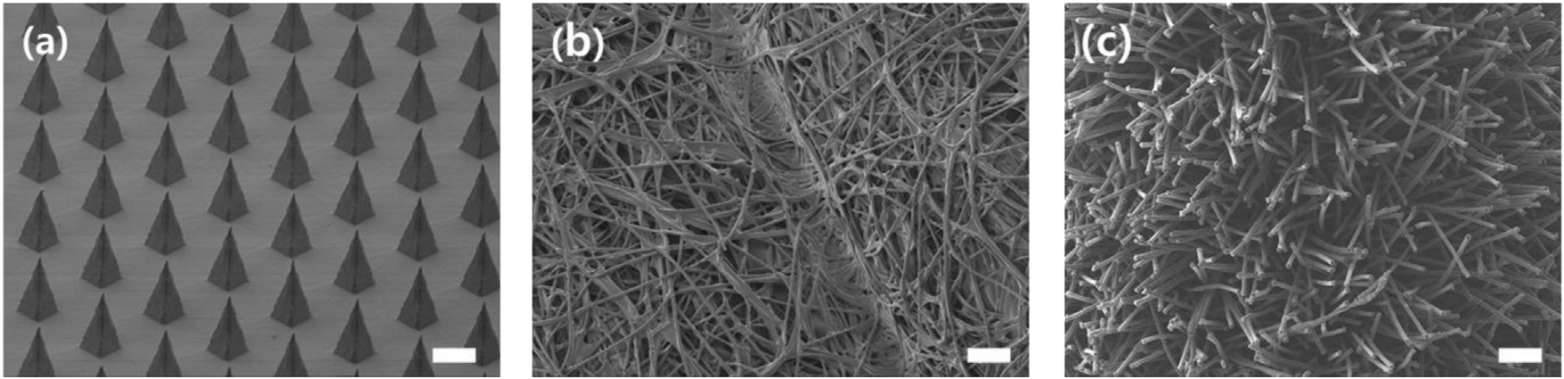
SEM images for comparison of morphology of MN swab and commercial swabs. From left: **(A)** MN swab, **(B)** Rayon swab (Isohelix^®^), **(C)** Nylon flocked swab (Copan eSwab^®^) (scale bars, 200 μm).

Figure 11 shows the morphology of an MN swab (S-N3-I3-S7) and two commercial swabs. The MN swab has structure that enables easy access to the lysis medium. The rayon swab consists of long, soft rayon fibers that are entangled each other. The nylon flocked swab has long, soft fibers. Because the fibers are soft, it is not easy to access the tissue below the mucosal surface.

##### Evaluation of Release Efficiency

Most commercial swabs, such as rayon swabs and nylon flocked swabs, have wrapped-around fibers or flocked fibers on a head. MN swabs, on the other hand, have an exposed structure so they can easily release large amounts of tissue sample and DNA into the lysis media. Recovering maximum amount of DNA is an important parameter for accurate analysis (Adamowicz et al., 2014).

**FIGURE 12:**
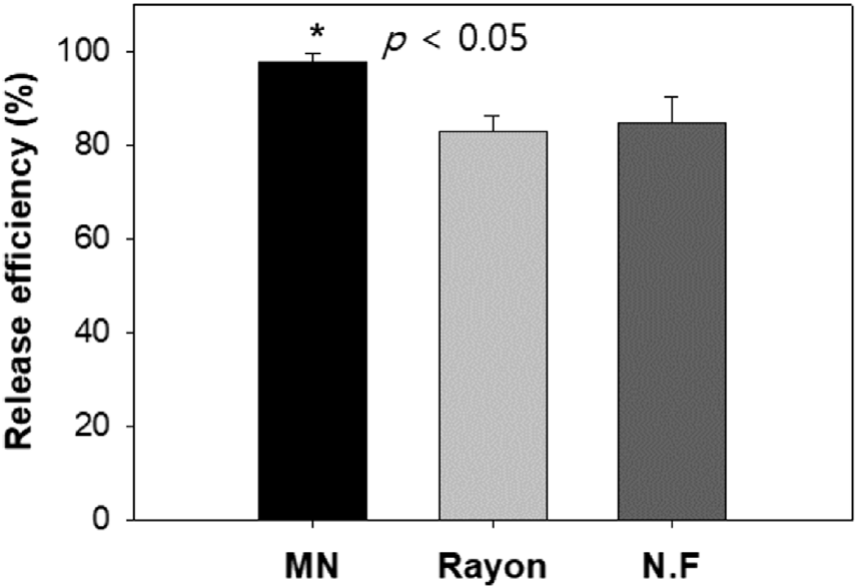
Comparison of release efficiency (%) observed for three swabs (the amount of DNA released into solution at 1 min from total amount of DNA collected by swabs): MN: microneedle swab, Rayon: rayon swab, N.F: nylon flocked swab (n = 4).

The release efficiencies achieved by the MN swab, the rayon swab, and the nylon flocked swab were 97.8, 82.8, and 84.8%, respectively, after putting the swab into the lysis buffer and vertexing it for 1 min. As can be seen in Figure 12, the release efficiency of the MN swab was higher than the release efficiency of the commercial swabs (*p* < 0.05). After 1 min, nearly all of the DNA was released from the MN swab. In real cases, the swab is not vortexed in lysis media. Thus, the role of the exposed structure of the microneedle is more important for accurate analysis in the field.

##### DNA Yield and Purity

**FIGURE 13:**
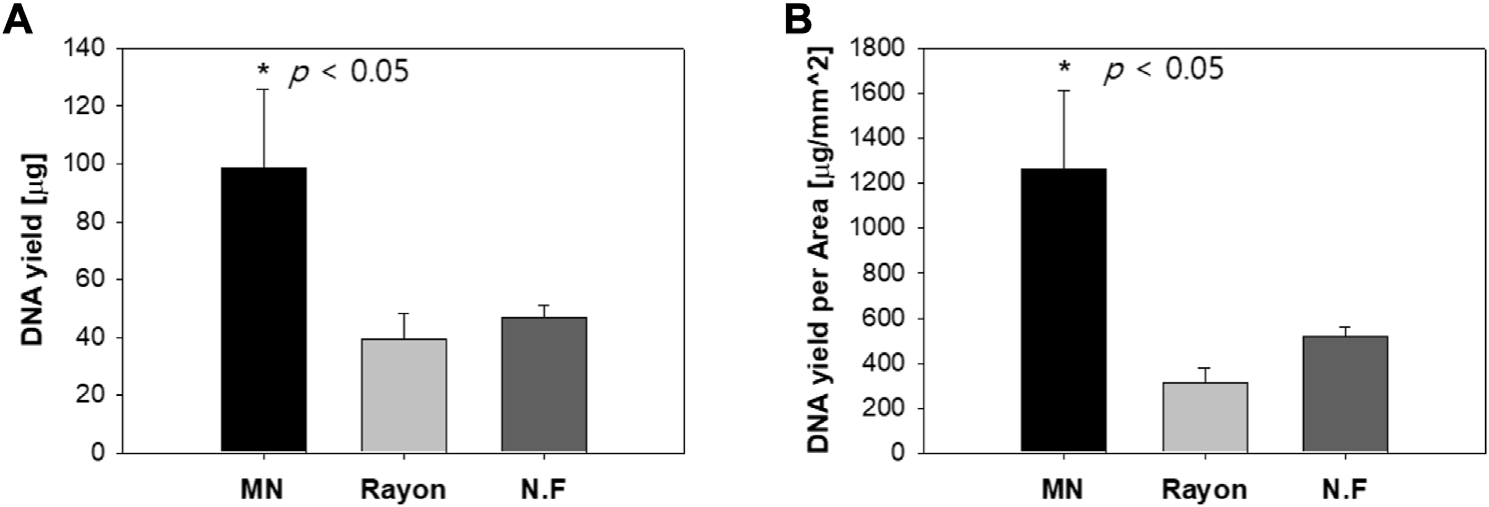
**(A)** DNA yield when *ex vivo* pig oral mucosa was swabbed 10 times using the S-N3-I3-S7 microneedle swab and two commercial swabs (rayon, nylon flocked swab). **(B)** DNA yield per unit area of swab head (MN: Microneedle; NF: Nylon Flocked). (n=5).

The DNA yield of the S-N3-I3-S7 MN swab and that of the commercial swab were compared. Because the area of each swab head was different, the DNA yield per area was also compared. After 10 times of swabbing, the average DNA yield was 98.5 μg with the MN swab, 39.6 μg with the rayon swab, and 46.7 μg with the nylon flocked swab (Figure 13A). That is, the DNA yield with the MN swab was greater than combined yield of the other two swabs (*p* < 0.05). In addition, Figure 13B shows the results obtained by dividing the DNA yield by the swab head area, indicating that the MN swab is better at collecting DNA than the two commercial swabs.

The quality of the DNA was just as important as the DNA yield for obtaining reliable data. At the absorbance of 260 nm, the nucleic acid was measured, and the contaminating protein or other contaminants of the sample were measured at the absorbance of 280 nm. The 260/280 ratio indicates the purity of the nucleic acid, which requires a value ranging from 1.6 to 2.1 (Ahmed et al., 2013). The average purity of the MN swab was 2.0, of the rayon swab was 1.8, and of the nylon flocked swab was 1.7. (Table 3). Thus, all three swabs were within acceptable ranges of DNA purity. Like other commercial swabs, MN swabs appear to provide reliable samples for accurate biomarker analysis.

**TABLE 3:** Comparison of DNA yield and purity of MN swab (S-N3-I3-S7) and two commercial swabs (Isohelix^®^ and Copan eSwab^®^).

Type of swab	DNA yield [μg]	DNA purity (260/280)
MN	98.5 ± 27.2	2.0 ± 0.1
Rayon (Isohelix^®^)	39.6 ± 8.8	1.8 ± 0.3
Nylon flocked (Copan eSwab^®^)	46.7 ± 4.2	1.7 ± 0.2

### Efficacy of Microneedle Swab in *in Vivo* Test

**FIGURE 14:**
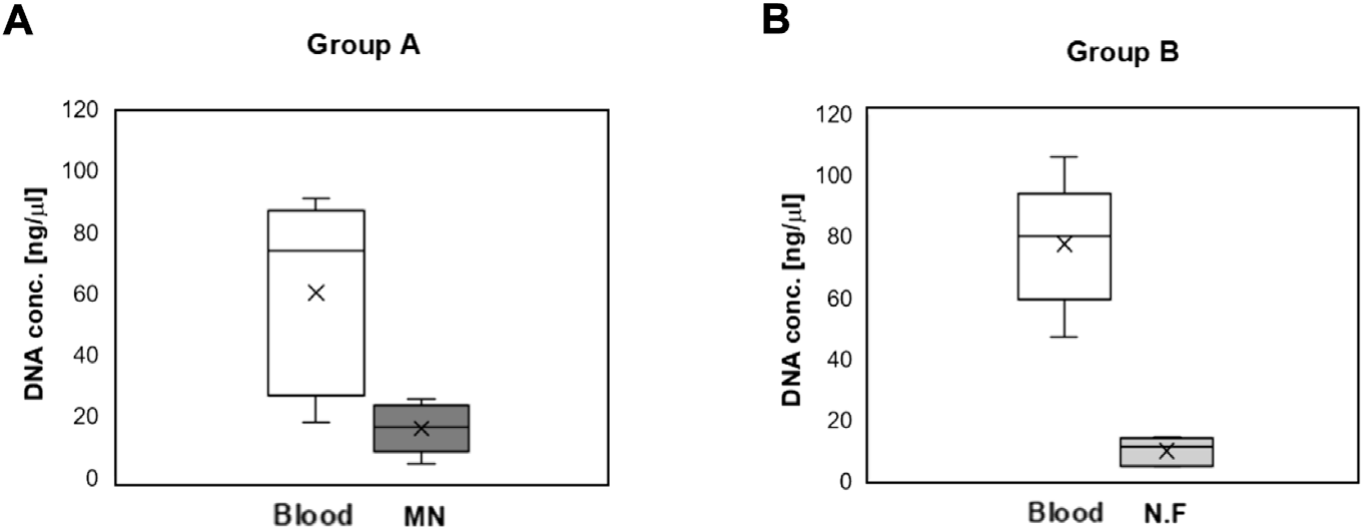
DNA concentration obtained from the blood and oral mucosa of mini-pigs. Oral mucosa was obtained by swabbing 10 times using a Z-N4-I2-S20 microneedle swab or a Copan eSwab^®^ nylon flocked swab (N.F) (n = 5). **(A)** Group A: The DNA concentration from a blood sample compared with that from a mucosa sample collected with a MN swab. **(B)** The DNA concentration from a blood sample compared with that from a mucosa sample collected with a N.F swab (Copan eSwab^®^) (MN: Microneedle swab, N.F: Nylon Flocked swab).

The DNA concentration and cycle threshold (Ct) values of samples obtained from the MN swab and the nylon flocked swab were compared with the values obtained from blood. A Z-N4-I2-S20 swab was used for the *in vivo* study. As seen in Figure 14, when samples were collected from a MN swab, the DNA concentration was 4–7 times lower than that collected from blood samples (*p* < 0.05). Specifically, the DNA concentration from blood was 60.3 ± 31.8 ng/μl and that from the MN swab was 16.1 ± 8.3 ng/μl (group A). The DNA concentration from blood was 77.4 ± 21.1 ng/μl and 9.9 ± 4.6 ng/μl from the nylon flocked swab. The DNA concentration of the samples obtained from both swabs was small compared to the concentration obtained from blood, but it is sufficient for PCR analysis. When the two swabs were compared, a higher average DNA concentration was obtained from the MN swab than from the nylon flocked swab.

When the values of DNA purity obtained by the three swabs (MN, rayon, nylon flocked) were compared, the value achieved by MN swab and the nylon flocked swab, was at 1.6 and 1.5 respectively. Both MN swabs and nylon flocked swabs showed lower purity value *in vivo* than those used in the *ex vivo* test; this was caused by the difficulty in controlling impurities of the oral cavity of the mini-pigs and the continuous movement of the mini-pigs during the experiment. In this experiment, there was no special food control, and, unlike human test, it was impossible to rinse the mouths of the mini-pigs before the experiment.

Next, real-time RT-PCR of the extracted DNA was performed. The intersection of the threshold at which the minimum reaction started and the amplification curve was the cycle threshold (Ct) value. This value indicated the relative amount of template DNA obtained from the sample. When the initial DNA amount was larger, the amplification curve appeared sooner. Therefore, lower Ct values indicate a higher amount of DNA (Kontanis and Reed, 2006).

**FIGURE 15:**
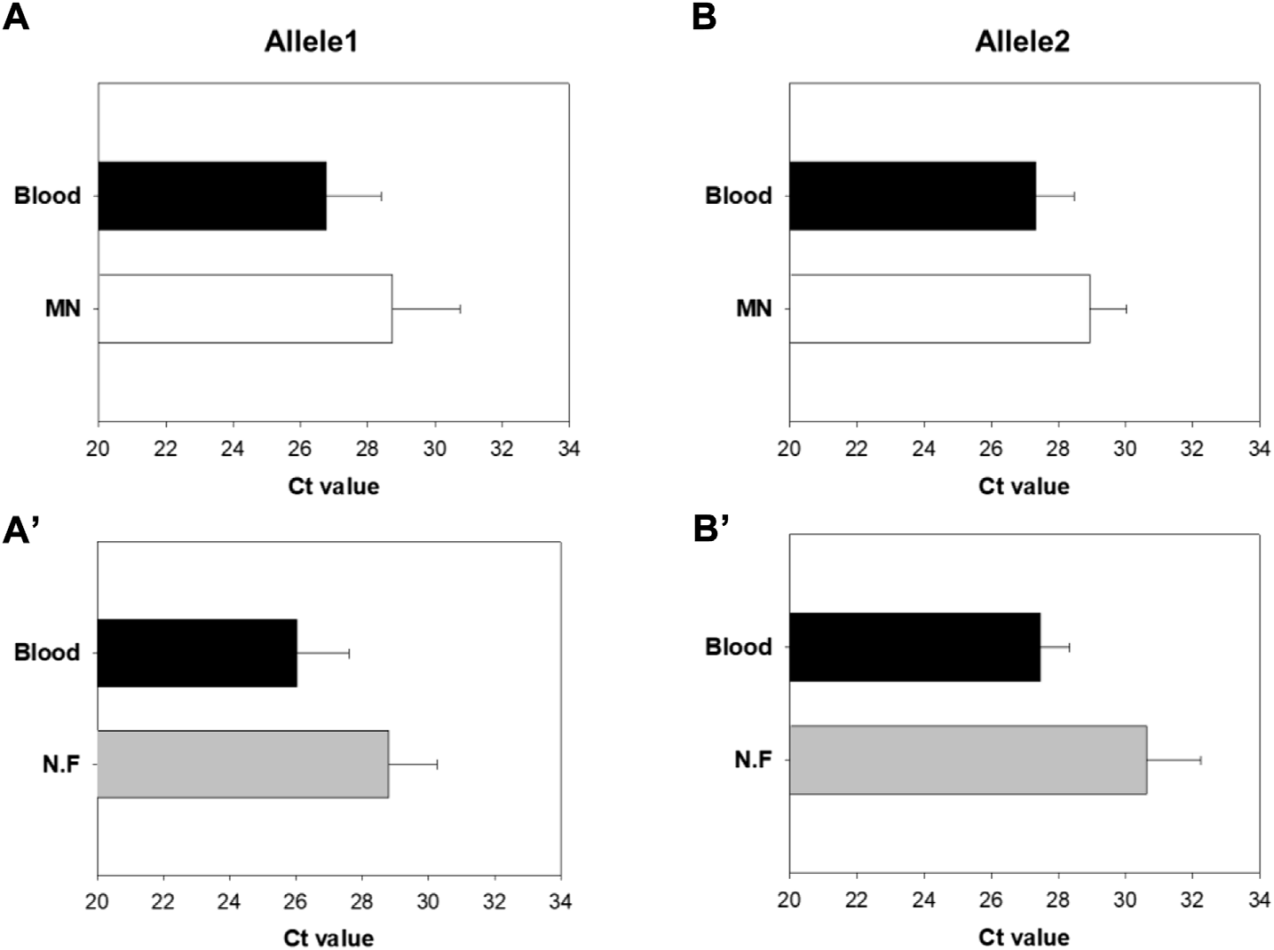
Comparison of cycle threshold (Ct) values of Groups A and B by real-time PCR of the extracted DNA **(A,Aʹ)** Results for Allele1 **(B,Bʹ)** Results for Allele2 (MN: Microneedle swab, N.F: nylon flocked swab) (n = 5).

Ct values of Group A and B were compared by real-time PCR using the DNA extracted from the mini-pigs (Figure 15). For Allele 1 of Group A, the mean Ct value of the blood sample was 26.8 and that of the MN swab sample was 28.7. For Allele 1 of Group B, the mean Ct value of the blood sample was 26.0 and that of the nylon flocked swab sample was 28.8. Likewise, for Allele 2 of Group A, the mean Ct value was 27.3 for blood and 28.9 for the MN swab sample. For Allele 2 of Group B, the mean Ct value was 27.5 for blood and 30.6 for the nylon flocked swab sample. These results show that the amount of DNA obtained by using the MN swab was higher than that obtained by using the nylon flocked swab. In animal experiments using mini-pigs, unexpected movements made it difficult to collect samples, which reduced efficacy. Therefore, we need to conduct efficacy studies through clinical tests on humans. In general, a buccal swab yields less DNA than blood, but the MN swab enabled us to obtain more DNA than commercial swabs.

## Conclusion

In this study, a microneedle swab was manufactured to obtain buccal samples from the oral cavity for DNA-based diagnostics, and its efficacy was evaluated. The very short length of the microneedles (250 μm) has the significant advantage of avoiding pain and irritation. The efficacy of the microneedle swab was evaluated according to the geometries of eight types of microneedles. Also, the efficacy of the MN swab was compared with two commercial swabs through *ex vivo* and animal experiments.

Polylactic acid and cyclic olefin copolymer have sufficient mechanical strength as material for a MN swab. For a MN swab, the number of microneedles and tip sharpness determine DNA yield. In addition, the zigzag pattern of the microneedles makes more swabbing lines on the oral mucosa compared to the straight pattern, resulting in increased DNA yield.

The MN swab showed improved efficacy compared to the commercial swabs in terms of DNA yield and DNA purity. Also, because the MN swab has an exposed structure, a large amount of DNA from the captured sample can be easily recovered into the lysis buffer. In addition, animal experiments demonstrated that the MN swab captured more DNA than commercial swabs.

Because MN swabs can be produced using a single injection molding process, it is possible to manufacture MN swabs more simply. The MN swab is easy to use, increases the DNA yield for diagnostics, and improves accuracy. Thus, the MN swab is an efficient and efficacious tool for conducting various biomarker analyses.

## Data Availability

The raw data supporting the conclusion of this article will be made available by the authors, without undue reservation.
