# Corrigendum: Development of a microneedle swab for acquisition of genomic DNA from buccal cells

**DOI:** 10.3389/fbioe.2022.1027776

**Published:** 2022-10-06

**Authors:** Yun-Seo Kim, Jeong Hyeon Kim, Woonsung Na, Gil-Hwan Sung, Seung-Ki Baek, Yun Kyoung Kim, Gyeong Ryeong Kim, Hae-Jin Hu, Jung-Hwan Park

**Affiliations:** ^1^ Department of Bionano Technology and Gachon BioNano Research Institute, Gachon University, Seongnam, South Korea; ^2^ Laboratory of Veterinary Virology, College of Veterinary Medicine, Chonnam National University, Gwangju, South Korea; ^3^ QuadMedicine R and D Centre, QuadMedicine Co, Ltd., Seongnam, South Korea; ^4^ Endomics, Inc., Seongnam, South Korea

**Keywords:** microneedles, swab, DNA yield, buccal mucosa, DNA purity

In the published article, there was an error in author’s affiliation information. Instead of “Jung-Hwan Park^1,3,^*”, it should be “Jung-Hwan Park^1,^*”.

The authors apologize for this error and state that this does not change the scientific conclusions of the article in any way. The original article has been updated.

