# Supplementary material for: Development of a Microneedle Swab for Acquisition of Genomic DNA From Buccal Cells
Source: Front Bioeng Biotechnol. 2022 Feb 17;10:829648. doi: 10.3389/fbioe.2022.829648 (PMC8895340; doi:10.3389/fbioe.2022.829648)
Supplement: Supplementary file 1 [file DataSheet1.DOCX]

**Supplementary Material**

| **Microneedle Swab Instruction** | |
| --- | --- |
| **1. Open microneedle swab**  Remove the microneedle swab from the wrapper by pulling the end of wrapper apart. | 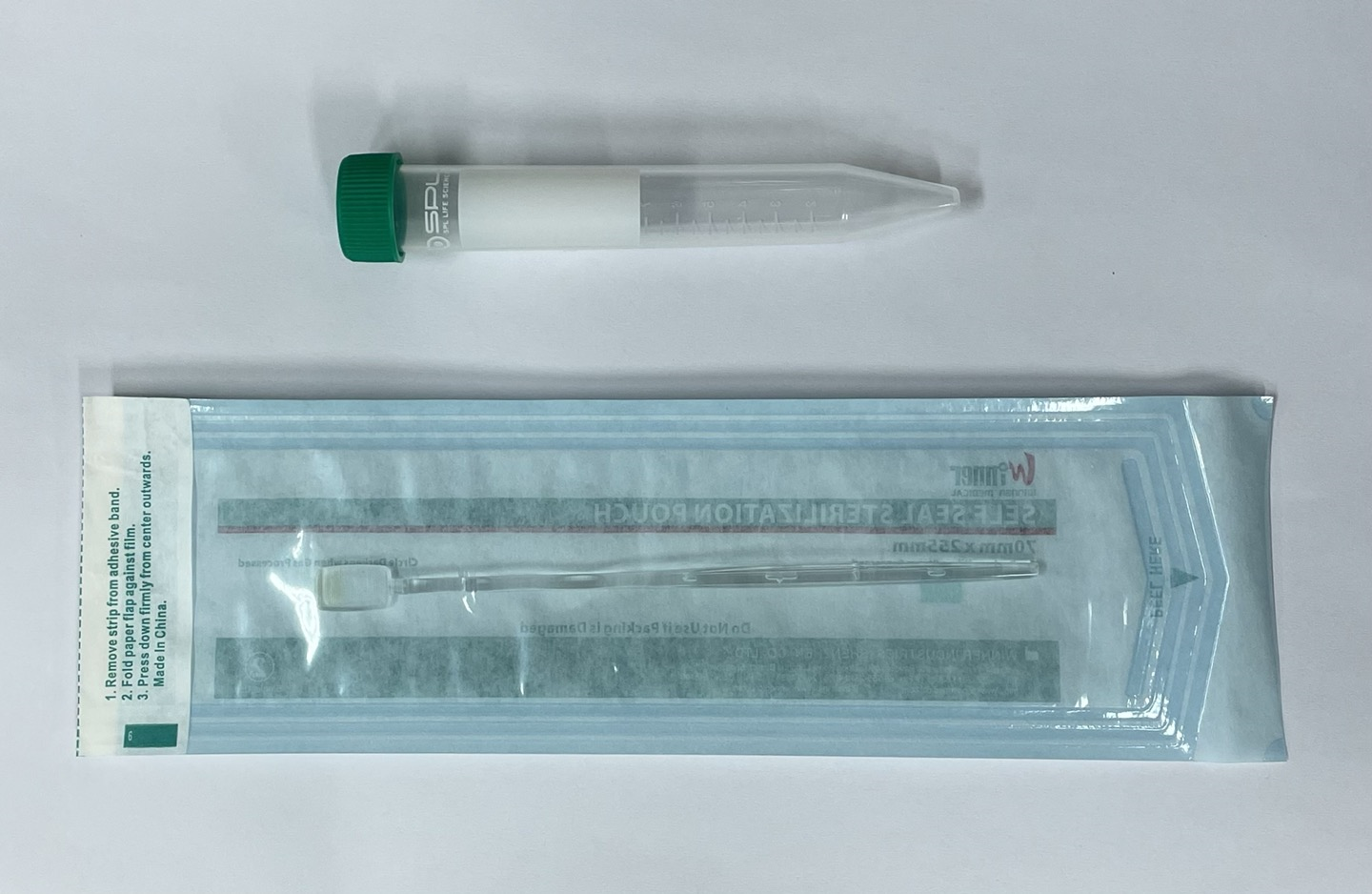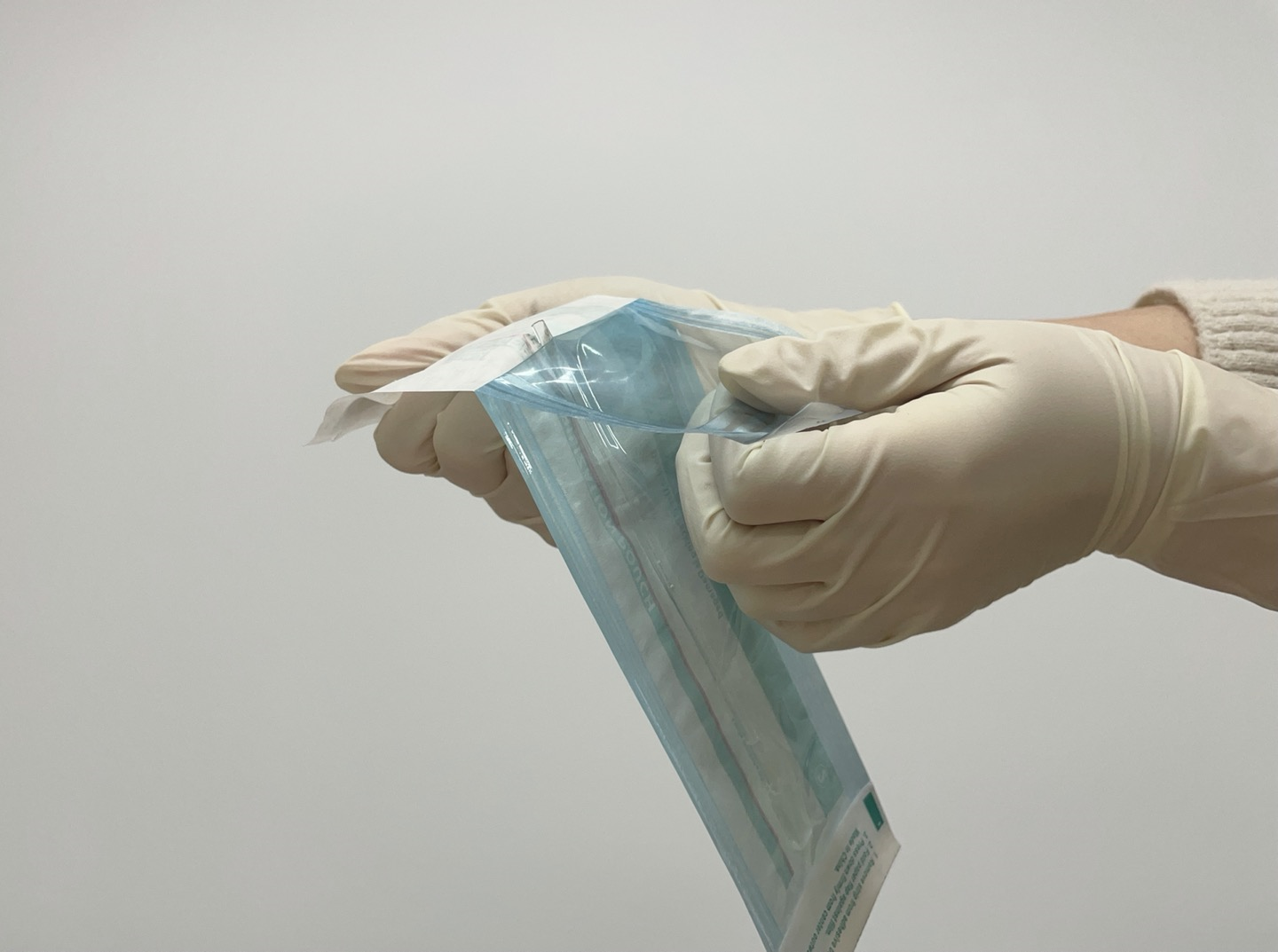 |
| **2. Swab buccal mucosa**  Gently insert the entire head of microneedle swab into the mouth until you feel contact on buccal mucus and swab in a back and forth motion 10 times in total. | 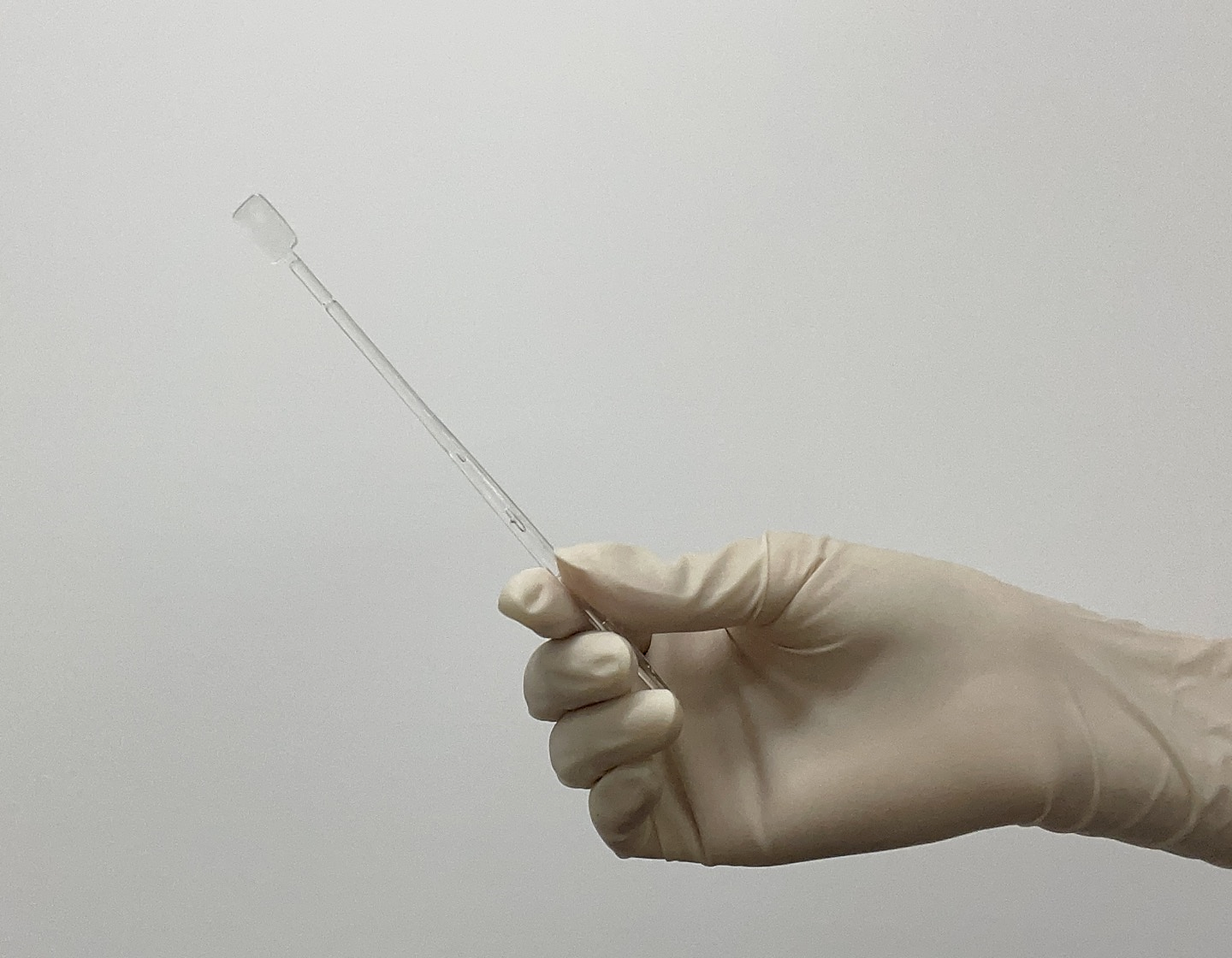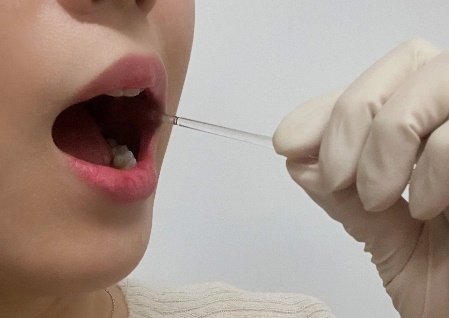 |
| **3. Put swab in tube**  Dip the swab into the prepared tube.  Once the head is at the bottom, break the swab handle at the top of the tube by bending back and forth. | 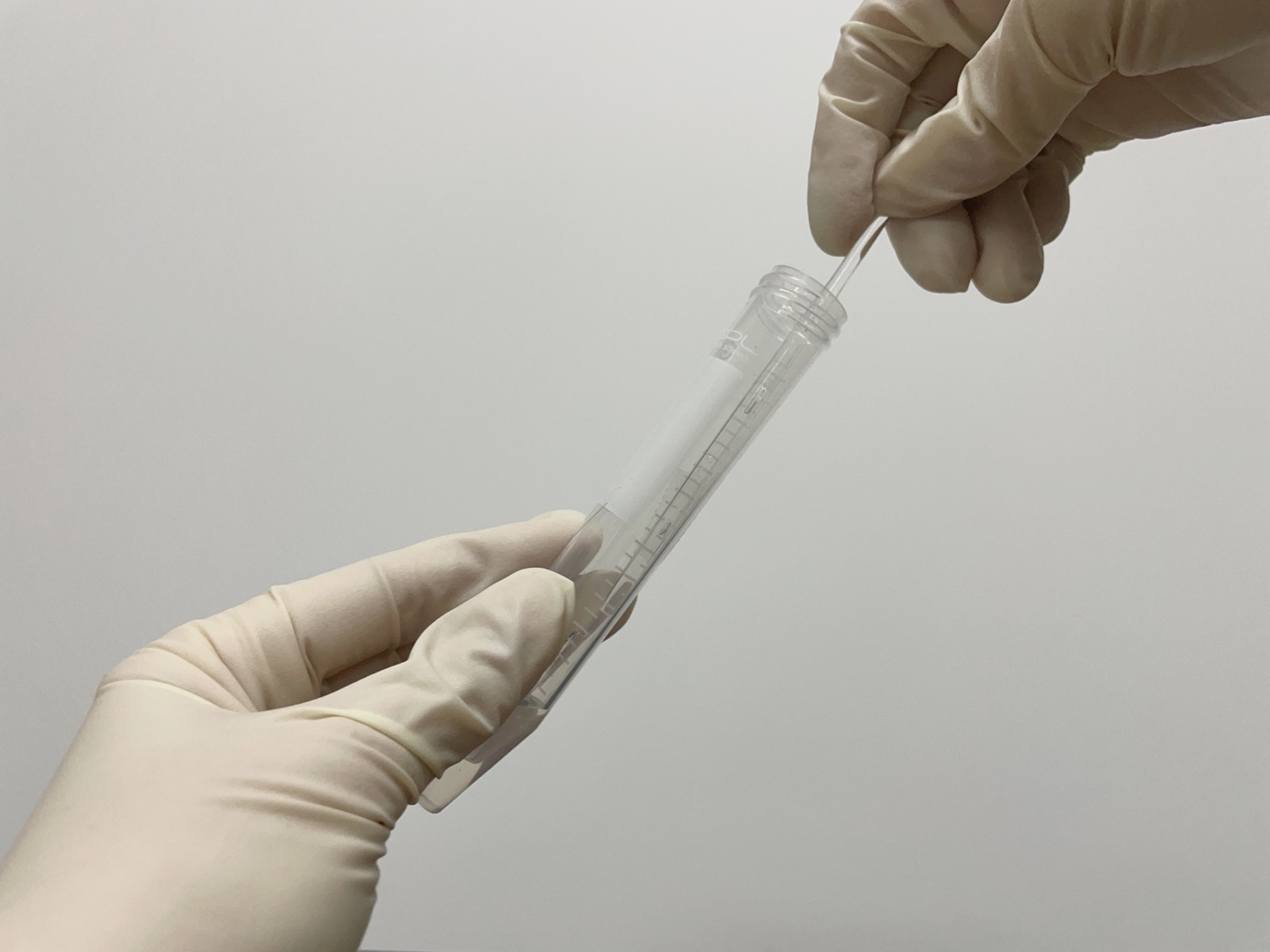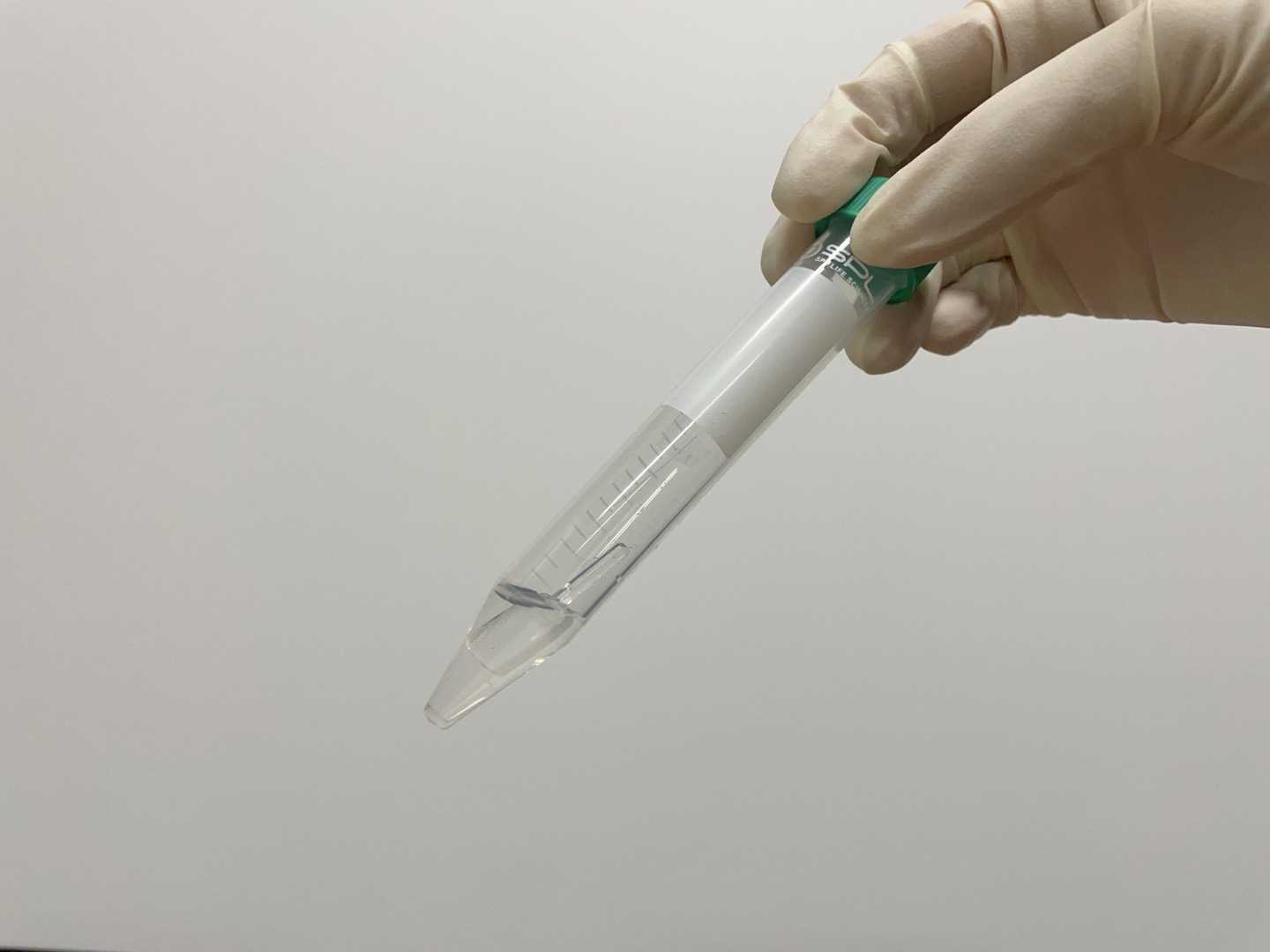 |

**Figure S1.** Microneedle swab collection instruction


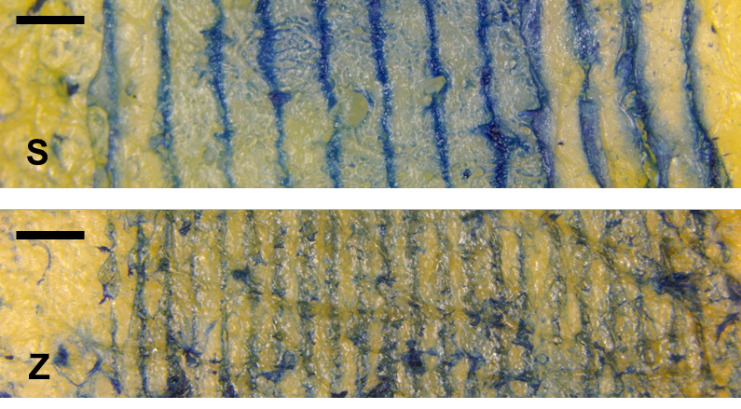


**Figure S2.** Optical images of porcine skin after swabbing with microneedle swab with straight pattern (S) and zigzag pattern (Z). The number of columns formed by microneedle swab with zigzag pattern (Z-N2-I4-S20) was twice that with microneedle swab with straight pattern (Z-N2-I4-S20) after swabbing the porcine skin once. Scale bar : 400 μm


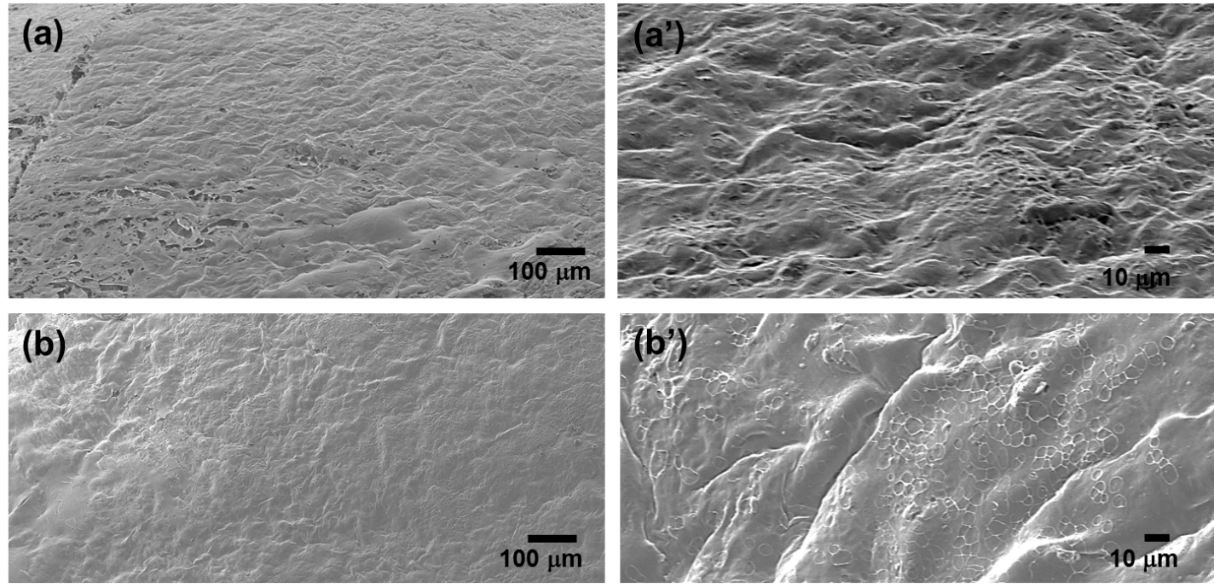


**Figure S3.** SEM images of the intact mucosal surface at 30 tilt angle and at (a) 100 and (a’) 500 of magnification, and SEM images of the 10 times swabbed mucosal surface at 30 tilt angle and at (b) 100 and (b’) 500 of magnification.

**Table S1. Swab protocol: DNA Purification from Buccal Swabs and Tissue protocol: DNA Purification from Tissues.**

| **Swab protocol: DNA Purification from Buccal Swabs.** | |
| --- | --- |
| 1 | The swab was put in the tube and 600 μl of PBS was added. |
| 2 | Then 20 μl of proteinase K and 600 μl of buffer AL were added to sample solution and mixed by vertexing. |
| 3 | After incubation at 56°C, 600 μl of ethanol was added. |
| 4 | The entire solution was applied to the spin column and centrifuged at 8000 rpm for 1 min. |
| 5 | Additional centrifugation was then performed at 8000 rpm after adding 500 μl of buffer AW1(wash) to the sample solution and centrifugation at 14000 rpm was applied after adding buffer AW2 to the sample solution. |
| 6 | 50 μl of elution buffer was applied, incubated for 1 minute, and centrifuged at 8000 rpm to obtain DNA. |

| **Tissue protocol: DNA Purification from Tissues.** | |
| --- | --- |
| 1 | The swab was placed in a solution of 420 μl of PBS, 180 μl of buffer ATL (tissue lysis), and 20 μl of proteinase K. |
| 2 | After incubation at 56°C, 600 μl of buffer AL was added. |
| 3 | After an additional incubation at 70°C for 10 min, 600 μl of ethanol was added. |
| 4 | The entire solution was applied to the column and centrifuged at 8000 rpm for 1 min. |
| 5 | Additional centrifugation was then performed at 8000 rpm after adding 500 μl of buffer AW1(wash) to the sample solution and centrifugation at 14000 rpm was applied after adding buffer AW2 to the sample solution. |
| 6 | Finally, 50 μl of elution buffer was applied, incubated for 1 minute, and centrifuged at 8000 rpm. |

*Source : QIAGEN QIAamp® DNA Mini and Blood Mini Handbook (5th Edition, Germany)

The difference between the two protocols is the buffer used to lyse the cells. The Swab protocol is for purifying DNA from a cellular-level specimen obtained from swabs, and the tissue protocol is for purifying DNA from a tissue-level specimen such as liver, kidney, and spleen. Therefore, to lyse relatively large amount of sample, a stronger lysis buffer like buffer ATL, should be used. In addition, an additional incubation process should be performed at 70°C for 10 minutes. In this experiment, since MN swab has a larger amount of sample compared to conventional commercial swabs and MNs swab obtain intact cells, two protocols are compared to find conditions for sufficient lysis.

| Group | Sample | Maximum value [ng/μl] | Minimum value [ng/μl] |
| --- | --- | --- | --- |
| Group A | Blood | 91.0 | 18.0 |
|  | MN | 25.7 | 4.5 |
| Group B | Blood | 106.0 | 47.3 |
|  | N.F | 14.6 | 4.9 |

**Table S2.** DNA concentration obtained from the blood and oral mucosa of mini-pigs. Oral mucosa was obtained by swabbing 10 times using a Z-N4-I2-S20 microneedle swab or a Copan eSwab® nylon flocked swab (N.F). (a) Group A : The DNA concentration from a blood sample compared with that from a mucosa sample collected with a MN swab. (b) The DNA concentration from a blood sample
